# Aging, light sensitivity and circadian health

**DOI:** 10.18632/aging.203806

**Published:** 2021-12-28

**Authors:** Sarah L. Chellappa

**Affiliations:** 1 Department of Nuclear Medicine, Faculty of Medicine and University Hospital Cologne, University of Cologne, Cologne, Germany

**Keywords:** aging, light sensitivity, sleep, circadian photosensitivity, behavioral interventions

The world is rapidly aging: In 2006, ~11% of the population worldwide were above 60 years, and it is predicted to double by 2050, resulting in more individuals above 60 years than those aged 0-14 [1]. This trend raises important questions, including what are the ramifications of aging for health and well-being.

Aging is often accompanied by sleep and circadian disruptions that may adversely affect quality of life. Compared to young individuals, those above 60 years typically experience increased wakefulness during sleep, early sleep and wake times and more sleep fragmentation [2]. Moreover, there is an association between healthy aging and disruption of endogenous circadian rhythms, as indexed by early timing and/or reduced amplitude of e.g., melatonin and core body temperature rhythms in older as compared to young individuals [2]. A key factor underlying these sleep and circadian changes in aging is reduced light sensitivity. Light elicits non-image-forming (NIF) effects primarily driven by intrinsically photosensitive retinal ganglion cells (ipRGCs) [3], by activating the photopigment melanopsin that is sensitive to light in the short-wavelength range (~490nm). Melanopsin-containing ipRGCs project to the suprachiasmatic nucleus (SCN, the circadian central pacemaker) through the retino-hypothalamic tract, and thereof to brain areas implicated in sleep-wake regulation [4].

**Figure 1:**
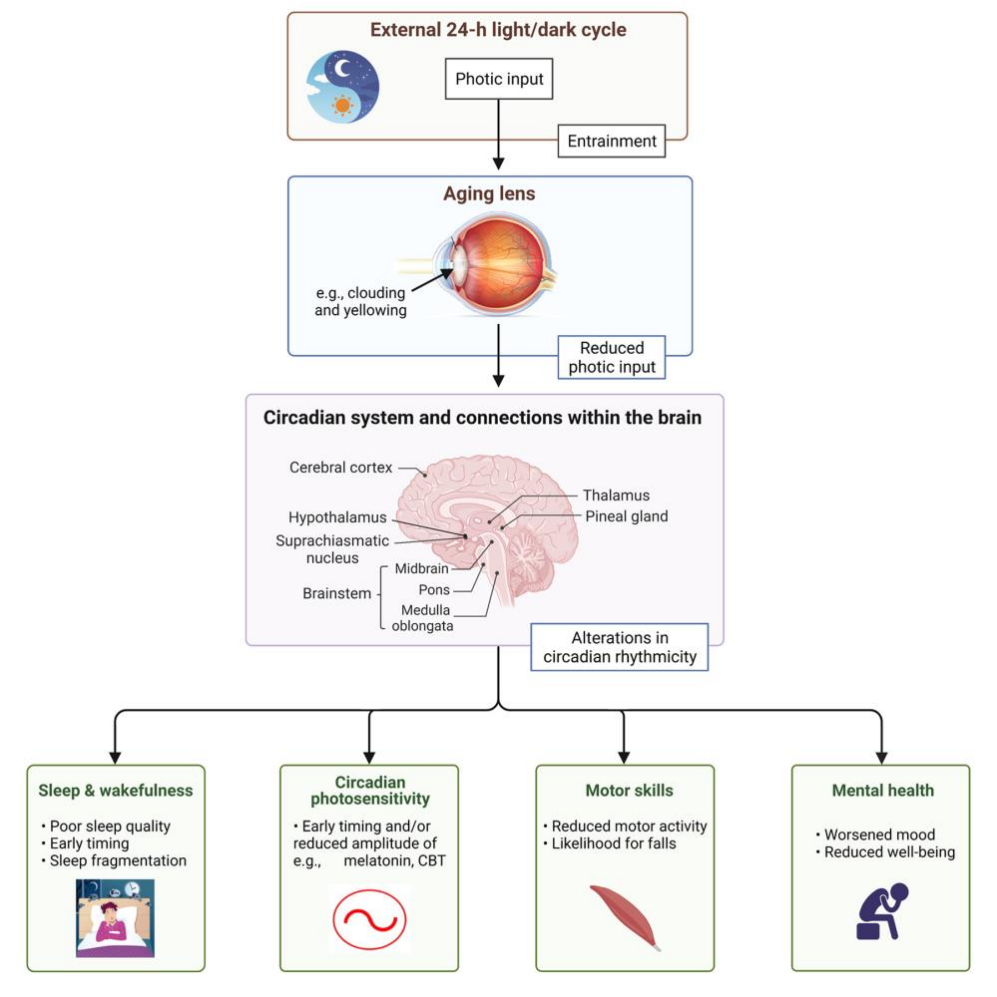
Conceptual framework for the effects of aging on light sensitivity within the eye and downstream.

Animal and human work indicate an age-related clouding and yellowing of the natural crystalline lens, increase in lens absorption, and reduction in pupil size and lens transmittance, as well a reduction in the number of photoreceptors and cells within the retina and the visual cortex [5]. Consequently, there might be a reduction in visual acuity, contrast sensitivity and visual processing speed, among others [5]. Importantly, the effects of aging lens extend beyond vision as the reduction of photic input to the SCN may elicit downstream effects, such as alterations in neuroendocrine and alerting responses to light, as well as changes in e.g., motor skills and mood (see Figure 1 for a schematic).

We therefore tested whether there are age-related changes in the NIF neuroendocrine and alerting responses to light [6]. Healthy unmedicated young (18-30 years) and older (55-80 years) individuals underwent a stringently controlled within-subject crossover study with three laboratory protocols separated by ~1-week. Each laboratory protocol included 3.5 h of dim-dark adaptation followed by 2 hours of acute polychromatic light at relatively low levels (~40 lx at horizontal gaze). Participants were exposed to light from a compact fluorescent light (CFL) source at 6500K (enriched in the short-wavelength light, 21.3 lx melanopic equivalent daylight illuminance, EDI), or one of two non-enriched in the short-wavelength light. The latter were either CFL at 2500K (13.3 lx melanopic EDI) or incandescent light source at 3000K (15.3 lx melanopic EDI). Thereafter, participants had a 30-min of dim light exposure (< 8 lx) followed by an 8-h sleep opportunity. We observed robust age-related effects in light sensitivity on melatonin suppression (a hallmark of circadian photosensitivity), subjective alertness, sustained attention and sleep slow-wave activity (a hallmark of sleep homeostatic regulation). In the healthy young individuals, acute evening light exposure at 6500K elicited more melatonin suppression, improved subjective sleepiness and sustained attention performance, and less slow-wave activity at the beginning of the sleep episode. In stark contrast, no differential light effects occurred in the healthy older individuals.

A common visual complaint in older adults including those without ocular diseases is the difficulty to see under low illumination levels, which can negatively affect the ability to perform daily tasks, increase the likelihood for falls, and decrease quality of life [7]. Our findings suggest that - at relatively low light intensities - there might be an age-related reduction in neuroendocrine and alerting responses to light. Healthy aging is not always associated with reduced light sensitivity on neuroendocrine function [2]. For instance, older individuals exposed to short-wavelength light at ~480nm show reduced melatonin suppression as compared to long-wavelength light at ~550nm, whereas no such differential effects in melatonin suppression have also been described, suggesting that circadian photosensitivity may still be preserved with aging [2]. Likewise, older individuals reported feeling more alert after exposure to short-wavelength light exposure (at 246 melanopic EDI or at 91.6 melanopic EDI) as compared to dim light [8]. As these previous studies used higher illuminances compared to our study, light exposure at lower intensities (typical in most indoor settings) might elicit dampened NIF light responses in older individuals. Therefore, increasing the amount of light exposure (particularly during the daytime) may help strengthen and boost the alerting responses to light. Ultimately, these evidence-based lighting solutions can provide an effective means to improve quality of life for an ever-growing older population.
